# Assessment of extracellular vesicle microRNAs detection in predicting benign and malignant pulmonary ground glass nodules

**DOI:** 10.3389/fmed.2025.1600429

**Published:** 2025-06-25

**Authors:** Ze Ji, Yufei Xing, Anyuan Zhong, Wensi Li, Suhang Cheng, Xing Pan, Minhua Shi

**Affiliations:** ^1^Department of Respiratory and Critical Care Medicine, The Second Affiliated Hospital of Soochow University, Suzhou, Jiangsu, China; ^2^Department of Respiratory and Critical Care Medicine, Suzhou Kowloon Hospital, Shanghai Jiaotong University School of Medicine, Suzhou, Jiangsu, China

**Keywords:** pulmonary ground glass nodules, extracellular vesicle microRNA, diagnostic value, sensitivity, liquid biopsy

## Abstract

**Objective:**

To assess extracellular vesicle microRNAs detection in predicting malignant and benign pulmonary ground glass nodules (GGNs).

**Methods:**

A retrospective study was conducted on 96 patients with pulmonary GGNs, hospitalized in Suzhou Kowloon Hospital and The Second Affiliated Hospital of Soochow University, Shanghai Jiaotong University School of Medicine between April 2021 and March 2022. Based on pathological diagnosis, 67 were in the malignant group and 29 in the benign group. Additionally, 50 healthy individuals from the same hospitals were enrolled as controls. Plasma exosomal microRNAs (miRNAs) levels were measured by quantitative reverse transcriptase polymerase chain reaction (qRT-PCR) in the three groups. The diagnostic value of candidate miRNAs was analyzed using receiver operating characteristic (ROC) curves.

**Results:**

Nodule size and proportion of pure GGNs were significantly higher in the malignant group than the benign group (*p* < 0.05). Expression of miR-17-5p and miR-1-3p was lower in malignant GGNs compared to benign ones, while miR-20b-5p, miR-9-5p, and miR-92a-2-5p were higher (*p* < 0.05). Compared to healthy controls, miR-1-3p and miR-17-5p were decreased, and miR-92a-2-5p, miR-9-5p, and miR-20b-5p were increased in malignant GGNs (*p* < 0.05). Among individual markers, miR-1-3p had the highest sensitivity and specificity. Sensitivity ranking: miR-1-3p > miR-20b-5p > miR-92a-2-5p > miR-9-5p > miR-17-5p; specificity ranking: miR-92a-2-5p > miR-17-5p > miR-1-3p > miR-9-5p > miR-20b-5p. Combining miR-1-3p and miR-92a-2-5p yielded an AUC of 1.000, with 100% sensitivity and specificity. In the healthy group comparison, miR-1-3p remained the most accurate single marker. Specificity ranking: miR-1-3p > miR-20b-5p > miR-92a-2-5p > miR-9-5p > miR-17-5p; sensitivity ranking: miR-1-3p > miR-17-5p > miR-92a-2-5p > miR-20b-5p > miR-9-5p.

**Conclusion:**

miR-1-3p, miR-9-5p, miR-17-5p, miR-20b-5p, and miR-92a-2-5p show promise as diagnostic biomarkers for GGN.

## Introduction

Lung cancer is one of the malignant tumors with high morbidity and mortality. In 2022, lung cancer was the most common cancer worldwide, causing nearly 2.5 million new cases and the leading cause of cancer deaths, with an estimated 1.8 million deaths (1). In 2015, the rate of incidence and mortality of lung cancer in China ranked first among malignant tumors, and the global incidence rate and mortality rate of lung cancer were about 11.4 and 18% (2). Studies have found that nodules with different densities have different possibilities of malignant risk. Compared with general solid nodules, Ground glass nodule (GGN) has a higher malignant risk. Because of its malignant potential and heterogeneity, GGN is particularly challenging to diagnose. Early studies mainly focused on the accurate CT findings of pulmonary nodules and the basic data of patients to build prediction models and evaluate the malignant risk.

However, a variety of CT features can help judge the nodules’ malignant risk, but most of them lean upon the subjective judgment and experience of clinicians. This requires the doctors to have practical clinical experience as well as theoretical understanding of the imaging methods and their proper interpretation. This makes the process prone to subjective judgment errors. One study showed that about 30% of nodules were misdiagnosed as malignant tumors by CT scanning (3). Some nodules are hard to identify as malignant or benign by CT imaging, peculiarly ground glass nodules with a diameter of about 1 cm. For such nodules, the guidelines recommend long-term CT follow-up observation or invasive operations such as tracheoscopy and minimally invasive surgery to further clarify the diagnosis. However, long-term CT follow-up increases the mental burden of patients, and lung tissue biopsy belongs to invasive examination. Some patients are not suitable for surgery, such as patients with severe emphysema and coagulation disorders. Therefore, it is necessary to combine relevant tumor markers to improve the diagnosis of malignant and benign lung nodules and avoid unnecessary surgical trauma. However, as most pulmonary nodules are in the initial stage of lung cancer pathology, the traditional tumor markers’ role in distinguishing malignant and benign pulmonary nodules are limited (4). Some nodules are hard to distinguish between benign and malignant by CT imaging, particularly ground glass nodules with a diameter of about 1 cm. For such nodules, long-term CT follow-up observations or invasive operations such as tracheoscopy or minimally invasive surgery to perform accurate diagnosis are recommended. However, long-term CT follow-up increases the burden on patients, and lung tissue biopsy is an invasive examination procedure. Some patient conditions like severe emphysema and coagulation disorders are not suitable for surgery. Therefore, it is important to combine relevant tumor markers to improve the diagnostics of and distinguish between malignant and benign lung nodules to avoid unnecessary surgical procedures. However, as most pulmonary nodules are in the initial phase of lung cancer pathology, the role of traditional tumor markers in distinguishing malignant and benign pulmonary nodules are limited.

Liquid biopsy is testing of tumor cells and tumor derivatives in blood, urine, saliva, and other body fluids, without the need for traditional histopathological analysis of biopsy. There have been tremendous advances in cancer diagnosis in recent years (5). Compared to the traditional tissue biopsy that requires invasive manipulation, liquid biopsy has the advantages of non-invasive and repeatability, which makes it easy to perform real-time monitoring of molecules changes in tumors (6). Additionally, liquid biopsy can detect biomarkers released from different tumor sites, comprehensively reflecting varieties in molecular images of malignant tumors’ solving the problem of tumor heterogeneity (7). In recent years, liquid biopsy biomarkers have been investigated for diagnosing lung nodules, mainly containing miRNA, cell-free DNA (cfDNA), circulating tumor cell (CTC) and autoantibodies (8). Among these biomarkers, exosomal miRNAs show great potential. This research aims to explore the potential application of extracellular vesicle microRNAs detection in predicting malignant and benign pulmonary ground glass nodules.

## Materials and methods

### Collection of patient clinical data

A retrospective analysis was performed on 96 cases with pulmonary ground glass nodules hospitalized in Suzhou Kowloon Hospital, Shanghai Jiaotong University School of Medicine and The Second Affiliated Hospital of Soochow University from April 2021 to March 2022. The pathological diagnosis showed that 29 patients in the benign pulmonary nodules group, mainly including 15 cases of non-specific inflammation, 8 cases of fibrous tissue hyperplasia, 3 cases of tuberculosis, 1 case of sclerosing pulmonary cell tumor, 1 case of pneumoconiosis and 1 case of organized pneumonia. There were 67 cases with malignant pulmonary nodules, containing 14 cases of adenocarcinoma *in situ*, 31 cases of microinvasive adenocarcinoma, 21 cases of invasive adenocarcinoma and 1 case of adenosquamous carcinoma. In addition, during the same period, 50 cases of healthy physical examination were collected as control group, and no lung related diseases were confirmed. This study was approved by the Ethics Professional Committee of The Second Affiliated Hospital of Soochow University.

### Instruments and reagents

Cfx-96 real-time fluorescent quantitative PCR instrument (Bio rad, United States); Exoquick™ reagent (American SBI company); Trizol reagent and total RNA Extraction Reagent (Qia gen, Germany); Reverse transcription kit, miRNA primer and QRT PCR reagent (genecopoeia, United States); Protein quantitative Kit (thermo company, United States); Rabbit monoclonal TSG101 protein (ab125011, 1/1000, Abcam, United Kingdom); Goat Anti-Rabbit IgG H&L IgG (ab6721, 1/2000, Abcam, United Kingdom); Rabbit monoclonal antibody CD9 protein: (ab236630, 1/1000, Abcam, United Kingdom).

### Extraction and identification of serum exosomes

1. Extraction of serum exosomes. 63 mL exoquick™ precipitant was added and the mixture was placed on ice in the refrigerator at 4°C for 30 min. The samples were centrifuged at 1,500 r/min for 30 min at 4°C. The supernatant was centrifuged for another 5 min to absorb the supernatant. 50 μL phosphate buffer was added, and the exosomes were shaken, blown, and suspended. The exosomes were used for further RNA extraction, exosome identification, or storage in the refrigerator at −80°C.
2. Serum exosomes were identified by transmission electron microscopy. First, the serum exosome samples to be identified were diluted at 50 ×, and 15 μL samples were placed on a carbon film copper mesh, carefully clamped on the copper mesh, and adsorbed for 1 min. The copper mesh was placed on the filter paper to absorb the excess sample. The prepared 2% uranyl acetate dye solution 15 μL was dropped for 1 min, and then the copper mesh was baked under the lamp for 10 min for observation.
3. Nanoparticle tracking analysis (NTA) technology. The exosomes were resuspended in phosphate buffer (1:10,000), and the samples were injected into the nanoparticle tracking analysis instrument. According to the characteristics of light scattering and Brownian motion, the particle size and concentration of the samples were analyzed. Duplicate samples were tested twice.
4. Identification of CD9 and tumor susceptibility gene 101 (TSG101) on the surface of exosomes by Western blot. Samples of extracted exosomes and serum proteins without exosomes were added to radioimmune precipitation assay (RIPA) protein lysates containing protease inhibitors for lysis. Protein quantification was performed according to the protein quantification kit. Proteins were separated on sodium dodecyl sulfate polyacrylamide gels and transferred to polyvinylidene difluoride membranes before blocking with fresh 5% skim milk powder. First and second antibodies to CD9 and TSG101 were incubated. The electrochemiluminescence method was used for color development and exposure.

### Extraction of serum exosomal RNA, reverse transcription and quantitative reverse transcriptase polymerase chain reaction

miR-16-5p was selected as internal reference for quantitative reverse transcriptase polymerase chain reaction (qRT-PCR) of each miRNA in serum exosomes. MiRNA specific amplification products were verified by cloning and sequencing. qRT-PCR primer sequences are proved in Table 1.

**Table 1:** Primer sequences of qRT-PCR.

miRNA	Sequences (5′-3′)
miR-17-5p	UCCCUGAGACCCUAACUUGUGAACAAGUUAGGGUCUCAGGGAUU
miR-133a-3p	UUUGGUCCCCUUCAACCAGCUGGCUGGUUGAAGGGGACCAAAUU
miR-1-3p	UGGAAUGUAAAGAAGUAUGUAUACAUACUUCUUUACAUUCCAUU
miR-20b-5p	UUGUGCUUGAUCUAACCAUGUAUGGUUAGAUCAAGCACAAUU
miR-92a-2-5p	UCUUUGGUUAUCUAGCUGUAUGAAUACAGCUAGAUAACCAAAGAUU
miR-9-5p	AACCCGUAGAUCCGAUCUUGUGCAAGAUCGGAUCUACGGGUUUU

### Statistical processing

SPSS 22.0 statistical software was employed for data processing. The 2^−ΔΔCt^ was used to calculate the relative expression of each miRNA molecule in each sample. The normal distribution’s measurement data were represented by mean ± standard deviation (x ± s), and the t test was used to conduct the statistical analysis. The non-normal distribution’s measurement data were represented by M (P25, p75), and the non-parametric Mann Whitney U test was used to conduct the statistical analysis. The counting data were exhibited as number and rate (%), and χ^2^ test was applied for comparison. Graphpad prism 8.0 software was used to draw scatter plots. Medcalc 9.6.4.0 was used to draw the receiver operating characteristic (ROC) curve and calculate the area under the curve (AUC). The diagnostic efficacy of differentially expressed miRNAs for GGN was calculated. *p* < 0.05 was considered statistically significant.

## Results

### General information and imaging data of two groups

In the comparison of basic information of the patients with pulmonary nodules between the two groups. No statistical difference was seen in gender, age, and other information between the two groups (*p* > 0.05). However, Compared with the benign group, the malignant group had larger average size of pulmonary nodules and (*p* < 0.01), as seen in Table 2.

**Table 2:** General information of the two groups.

Items	Malignant group (*n* = 67)	Benign group (*n* = 29)	χ^2^/t	*p*-value
Gender			0.07	0.72
Male	39 (58.20)	16 (55.20)		
Female	28 (41.80)	13 (44.80)		
Age (years)	51.9 ± 12.8	51.1 ± 11.4	0.29	0.77
Size of pulmonary nodules (mm)	13.4 ± 1.5	8.3 ± 1.3	15.89	<0.01
Types of ground glass nodules			3.64	0.06
Pure ground glass nodules	44 (65.67)	13 (44.83)		
Mixed ground glass nodules	23 (34.33)	16 (55.17)		
Smoking history			0.20	0.65
Yes	47 (69.12)	19 (65.52)		
No	20 (29.85)	10 (34.48)		
Hypertension			0.09	0.75
Yes	37 (55.22)	17 (58.62)		
No	30 (44.78)	12 (41.38)		
Diabetes			0.34	0.55
Yes	25 (37.31)	9 (31.03)		
No	42 (62.69)	20 (68.97)		
Medication use			1.66	0.19
Yes	35 (52.24)	11 (37.93)		
No	32 (47.76)	18 (62.07)		

### Identification of exosome markers in serum of lung adenocarcinoma and healthy people

**Figure 1:**
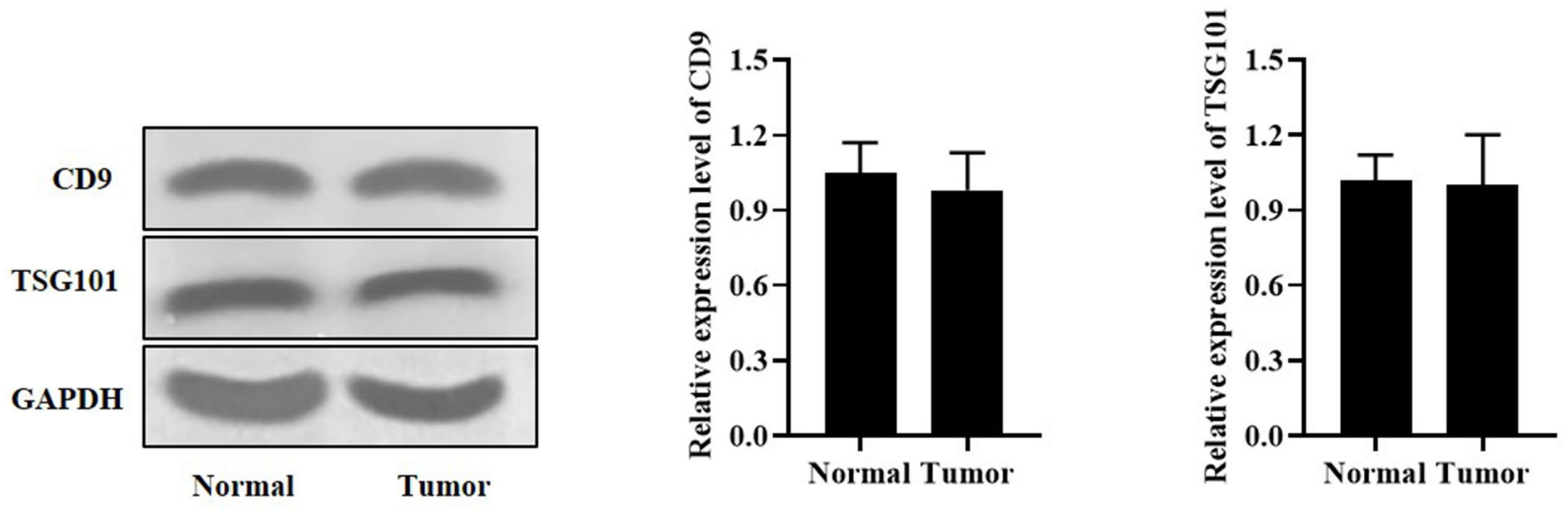
Western blot analysis of CD9 and TSG101 proteins in the serum of tumor patients and healthy subjects.

As displayed in Figure 1, the expression levels of CD9 and TSG101 proteins were significantly present in the serum of tumor patients and healthy subjects.

### Distribution of six candidate miRNAs in benign lung nodule and malignant lung nodule group

**Figure 2:**
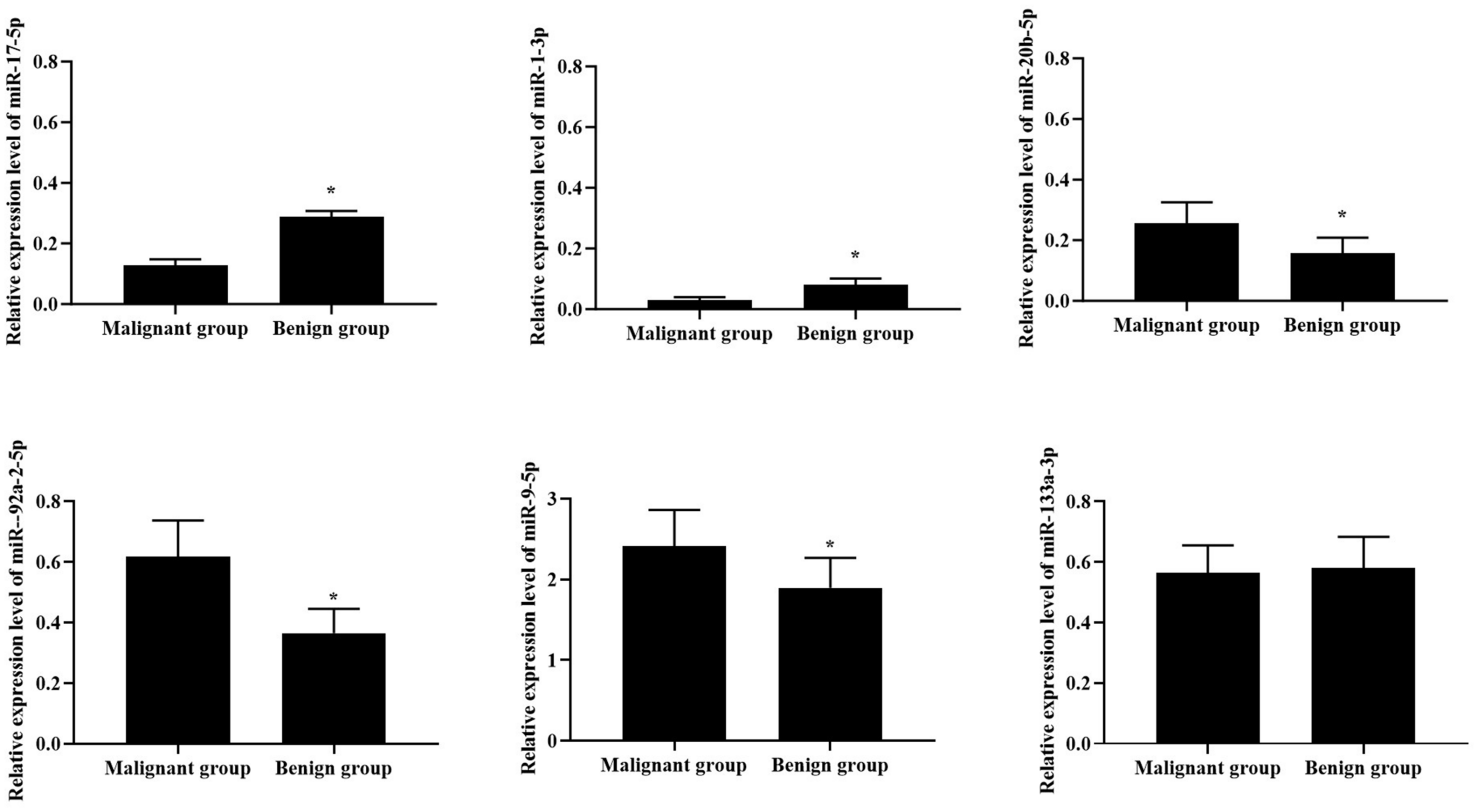
Distribution of six candidate miRNAs in benign and malignant lung nodule group. ^*^p < 0.05, in comparison with malignant group.

The results displayed that there were statistical differences in the expression levels of miR20b-5p, miR-9-5p, miR-1-3p, miR-17-5p and miR-92a-2-5p between malignant and benign pulmonary nodules (*p* < 0.05), Among them, the expression of miR-17-5p and miR-1-3p in malignant GGN group was lower than benign GGN group, while the expression of miR-92a-2-5p, miR-9-5p and miR-20b-5p in malignant GGN group was higher than benign GGN group, as shown in Table 3 and Figure 2. All these results suggest that miR-17-5p, miR-1-3p, miR-92a-2-5p, miR-9-5p and miR-20b-5p may be potential biomarkers for differentiating benign and malignant pulmonary nodules.

**Table 3:** Distribution of six candidate miRNAs in benign and malignant lung nodule group.

miRNA	Malignant GGN group	Benign GGN group	*p*-value
miR-17-5p	0.128 ± 0.02	0.288 ± 0.02	<0.05
miR-1-3p	0.030 ± 0.01	0.081 ± 0.02	<0.05
miR-20b-5p	0.256 ± 0.07	0.158 ± 0.05	<0.05
miR-92a-2-5p	0.617 ± 0.12	0.356 ± 0.08	<0.05
miR-9-5p	2.411 ± 0.45	1.896 ± 0.37	<0.05
miR-133a-3p	0.564 ± 0.091	0.581 ± 0.102	>0.05

### Distribution of six candidate miRNAs in malignant lung nodule and healthy group (no nodule control + benign lung nodule group)

**Figure 3:**
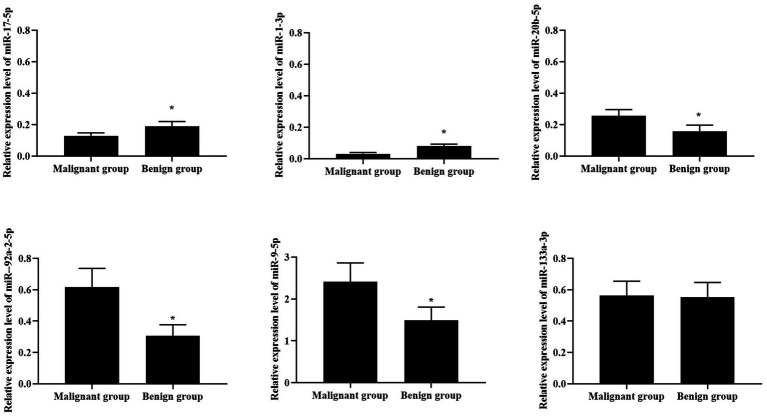
Distribution of six candidate miRNAs in malignant pulmonary nodule and healthy group. ^*^p < 0.05, compared with malignant group.

To further verify the role of candidate miRNAs in the screening of malignant GGN and to apply them in clinical practice, we further divided the patients into the malignant GGN group and the healthy group. The healthy group included the benign GGN group and the control group without pulmonary nodules. The results showed that the expression of miR-92a-2-5p, miR-1-3p, miR-20b-5p, miR-17-5p and miR-9-5p in malignant GGN group and healthy group was statistically different (*p* < 0.05). Among them, the expression of miR-17-5p and miR-1-3p in malignant GGN group was lower than healthy group (0.127 vs. 0.180, 0.030 vs. 0.081, FM), while the expression of miR-20b-5p, miR-9-5p and miR-92a-2-5p was higher in malignant GGN group (0.230 vs. 0.157, 0.617 vs. 0.307, 2.411 vs. 1.785, FM), as seen in Figure 3 and Table 4. All these results suggest that miR-17-5p, miR-1-3p, miR-92a-2-5p, miR-9-5p and miR-20b-5p may be potential biomarkers to distinguish patients with malignant pulmonary nodules.

**Table 4:** Distribution of six candidate miRNAs in malignant pulmonary nodule and healthy group (benign pulmonary nodule + no nodule control group).

miRNA	Malignant GGN group	Health group	*p*-value
miR-17-5p	0.128 ± 0.02	0.190 ± 0.03	<0.05
miR-1-3p	0.030 ± 0.01	0.081 ± 0.012	<0.05
miR-20b-5p	0.256 ± 0.04	0.157 ± 0.04	<0.05
miR-92a-2-5p	0.617 ± 0.12	0.307 ± 0.07	<0.05
miR-9-5p	2.411 ± 0.45	1.485 ± 0.32	<0.05
miR-133a-3p	0.564 ± 0.091	0.553 ± 0.094	>0.05

### Diagnostic ability of miR-9-5p, miR-1-3p, miR-20b-5p, miR-17-5p and miR-92a-2-5p for benign and malignant pulmonary nodules

The basic concept of ROC curve is to regard the sensitivity and specificity as a continuous process, and use a curve to describe the performance of the diagnostic system. The principle of drawing the curve is to calculate the corresponding sensitivity and specificity at different boundary points of continuous variable, and then plot the curve of true positive rate and false positive rate with sensitivity as the vertical coordinate and 1-specificity as the horizontal coordinate. AUC is the area under the ROC curve, which is between 0.1 and 1. As a numerical value, the prediction accuracy of the model can be intuitively evaluated. The larger the AUC value, the higher the prediction accuracy.

**Figure 4:**
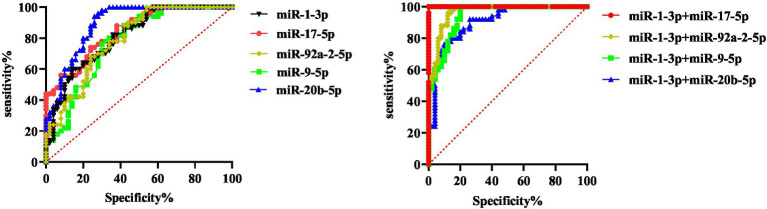
Diagnostic ability of 5 miRNAs for benign and malignant pulmonary nodules.

Based on the data of benign and malignant pulmonary nodules, we determined whether the malignant pulmonary nodules were positive, drew the ROC curve, and then evaluated the diagnostic capabilities of these five miRNAs used alone or in combination for malignant pulmonary nodules. The results showed that when the five miRNAs were used for diagnosis alone, miR-1-3p had the highest sensitivity and specificity, and the specificity from high to low was as follows: miR-92a-2-5p, miR-17-5p, miR-1-3p, miR-9-5p, and miR-20b-5p. The sensitivity from high to low was as follows: miR-9-5p, miR-1-3p, miR-20b-5p, miR-17-5p, miR-92a-2-5p. We separately combined other indicators with miR-1-3p for diagnosis, and the results displayed that through the combined diagnosis of miR-1-3p and miR-92a-2-5p, the AUC value for malignant pulmonary nodules was 1.000, and both the sensitivity and specificity reached 100%. The combination of other indicators could also correspondingly enhance the diagnostic ability (Figure 4; Table 5). The results show that exosomal miR-1-3p and miR-92a-2-5p have extremely high application value in diagnosing malignant and benign pulmonary nodules.

**Table 5:** Diagnostic ability of five miRNA for malignant and benign pulmonary nodules.

MiRNAs	AUC	95% CI	*p*-value	Optimum value	Sensitivity	Specificity
miR-l-3p	0.8416	0.7620–0.9418	<0.001	≤0.1745	0.8800	0.7062
miR-20b-5p	0.7724	0.6920–0.9020	<0.001	≥0.0445	0.8220	0.6534
miR-92a-2-5p	0.8914	0.6696–0.8876	<0.001	≥0.1605	0.8000	0.8000
miR-9-5p	0.8084	0.6045–0.8413	0.015	≥0.3995	0.7400	0.6600
miR-17-5p	0.7908	0.4815–0.7613	0.036	≥1.765	0.6200	0.7600

### Diagnostic ability of miR-92a-2-5p, miR-1-3p, miR-9-5p, miR-20b-5p and miR-17-5p for malignant solitary pulmonary nodule and healthy group

Based on the data from the malignant solitary pulmonary nodule (SPN) group and the healthy group (the benign pulmonary nodule group + the no-nodule control group), ROC curves were plotted to evaluate the ability of five types of miRNAs, either individually or in combination, for the early diagnosis of lung cancer, and the positive results of screening out malignant SPNs were used as the evaluation basis.

**Figure 5:**
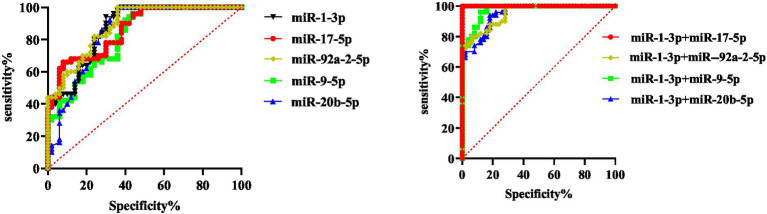
Diagnostic value of five miRNAs in healthy people and patients with malignant pulmonary nodules.

The results showed that when the five miRNAs were used for diagnosis alone, miR-1-3p had the highest sensitivity and specificity, and the specificity from high to low was as follows: miR-1-3p, miR-20b-5p, miR-92a-2-5p, miR-9-5p, and miR-17-5p. The sensitivity from high to low was as follows: miR-1-3p, miR-17-5p, MiR-92a-2-5p, miR-20b-5p, miR-9-5p. In the combined diagnosis of these two types of miRNAs, the combined sensitivity of miR-20b-5p and miR-1-3p could reach 100%, and the specificity was 93.0%. When miR-1-3p was combined with miR-92a-2-5p, the specificity could reach 100% and the sensitivity was 97.9%. When these three types of miRNAs were used for combined diagnosis, the sensitivity and specificity of miR-1-3p, miR-92a-2-5p and miR-17-5p (or miR-20b-5p) could all reach 100% (Figure 5). The results show that the combination of the two exosomal miRNAs can be used to screen malignant GGN in the population.

## Discussion

With the wide application of low-dose spiral CT, the detection rate of pulmonary GGN has significantly increased (9). However, the false positive rate of low-dose spiral CT in screening for pulmonary nodules is relatively high, which brings great psychological pressure to patients and leads to many unnecessary invasive diagnostic and therapeutic procedures. Therefore, improving the diagnostic efficiency of malignant pulmonary nodules has become one of the urgent problems to be solved.

Exosomal miRNAs have become a research hotspot in the field of liquid biopsy in recent years and have achieved preliminary research results in lung cancer diagnosis. Zheng et al. analyzed the plasma exosomal miRNAs spectra of 46 patients with early non-small cell lung cancer (NSCLC) and 42 healthy individuals using miRNA SEQ technology, successfully screening out four miRNA combinations (let-7b-5p, let-7e-5p, miR-23a-3p, and miR-486-5p). This combination demonstrated a sensitivity of 80.25% and a specificity of 92.31% in the diagnosis of NSCLC (10). Besides, four exosomal miRNAs (hsa-miR-1269a, hsa-miR-210-5p, hsa-miR-205-5p and hsa-miR-9-3p) have been proven to play a positive role in differentiating patients with NSCLC from healthy individuals. The AUC value in the training group was 0.915, and the AUC value in the validation group was 0.878 (11). Additionally, a study used qRT-PCR technology to detect serum exosomal miRNAs in 110 patients with lung adenocarcinoma and healthy individuals. The results showed that the concentration of miR-1290 in patients with lung adenocarcinoma was significantly higher than that in the healthy control group. The researchers also compared miR-1290 with the traditional lung cancer tumor markers-carcinoembryonic antigen (CEA), cytokeratin-19 fragment (CYFRA21-1), and neuron-specific enolase (NSE) using the ROC curve. They found that miR-1290 had the highest sensitivity and specificity in diagnosing lung adenocarcinoma, with the sensitivity and specificity being 80.0 and 96.7%, respectively (12).

In this study, we included 96 patients with solitary pulmonary nodule (SPN) and 50 healthy controls. The average size of malignant and benign SPNs was 12.4 mm and 9.3 mm, respectively, which were used to verify the diagnostic value of plasma exosomal miRNAs in pulmonary nodules with a diameter of approximately 1 mm. Through qRT-PCR analysis, five plasma exosomal miRNAs (miR-1-3p, miR-17-5p, miR-9-5p, miR-20b-5p and miR-92a-2-5p) were identified as potential biomarkers of malignant SPNs. Among the single indicators, miR-1-3p had the best diagnostic effect, with a sensitivity of 89.6% and a specificity of 100%. When it was combined with miR-92a-2-5p, the specificity and sensitivity reached 100%, indicating that this combination could effectively distinguish malignant and benign SPNs. In addition, we further analyzed the diagnostic value of miR-1-3p combined with other miRNAs in detecting early lung cancer. Two sets of miRNA combinations (miR-92a-2-5p, miR-1-3p and miR-17-5p) and (miR-92a-2-5p, miR-1-3p and miR-20b-5p) were identified as potential indicators of early lung cancer, with sensitivity and specificity of 100%. It is likely that miR-1-3p and miR-92a-2-5p are involved in the occurrence and development of lung cancer. However, their roles in the tumor formation process still require further investigation.

Among the five miRNA indicators in this study, miR-1-3p has been reported to inhibit the proliferation of human NSCLC cells by negatively regulating its target gene cyclin dependent kinase 9 (CDK9), thus promoting the apoptosis of NSCLC cells (13). CDK9 is one of the most important transcription regulatory members of the CDK family (14). CDK9 regulates a plethora of cellular functions including proliferation, apoptosis, cell cycle regulation, DNA damage repair and metastasis (15). Targeting CDK9 is considered as an effective treatment for many cancers, such as triple negative breast cancer (16), small cell lung cancer (17) and colorectal cancer (18). Leidinger et al. confirmed the expression level of miR-17-5p was relatively low in lung squamous cell carcinoma, and speculated that its target gene was ribonucleotide reductase M2 (RRM2) through gene analysis. RRM2 is a part of nucleotide reductase, catalyzes the inhibition of ribonucleotides, yielding deoxyribonucleotides (19). Previous studies have revealed that RRM2 plays a vital role in promoting cell proliferation, migration, and invasion, while inhibiting cell apoptosis (20). RRM2 has been reported to be upregulated in many types of cancer and has been implicated in tumor progression, including NSCLC (21, 22). As reported previously, miR-17-5p inhibits the expression of RRM2, thereby exerting an anti-tumor effect (23). miR-20b-5p over-expression can inhibit the killing ability of NK cells against lung adenocarcinoma cells and promote the growth of lung cancer by inhibiting the expression of SHMT1 (24). Li et al. (25) reported that the expression of miR-9-5p in the lung tissues of NSCLC patients was significantly higher than that in the adjacent normal lung tissues, and miR-9-5p accelerated the metastasis, invasion and proliferation of lung cancer cells by targeting transforming growth factor beta receptor 2 (TGFBR2). Although the expression level of miR-92a-2-5p in lung adenocarcinoma tissues has been confirmed to be significantly higher than that in normal lung tissues in previous experiments (26), the specific mechanism has not been clearly reported. Consistent with the above studies, our research results indicated that the expression of miR-1-3p and miR-17-5p in malignant pulmonary nodules was lower than those in benign pulmonary nodules and the healthy control group, while the expression of miR-20b-5p, miR-9-5p and miR-92a-2-5p was higher in malignant pulmonary nodules.

Our study has some limitations. Firstly, our sample size is relatively small, which may lead to deviations between the data results and the actual values. Secondly, our research was a single-center study, and the sample was not representative, which may not accurately reflect the characteristics of a broader population. In addition, the formation of pulmonary nodules may be related to lung inflammation or infection (27, 28). During an inflammatory or infectious state, the local environment of lung tissue changes, which may lead to alterations in the miRNA expression profile, and these changes may affect the growth, development or characterization of pulmonary nodules (29, 30). However, our study did not perform subgroup analysis to detect miRNA expression in patients with benign pulmonary nodules. Therefore, more multi-center and large-scale studies as well as subgroup analysis of miRNA expression in patients with benign pulmonary nodules should be conducted in the future to further verify our findings.

To sum up, our study demonstrates that miR-17-5p, miR-1-3p, 303 miR-92a-2-5p, miR-20b-5p, and miR-9-5p can be used as potential biomarkers for diagnosing GGN.

## Data Availability

The datasets presented in this study can be found in online repositories. The names of the repository/repositories and accession number(s) can be found in the article/supplementary material.
